# Recent Progresses in Nanobiosensing for Food Safety Analysis

**DOI:** 10.3390/s16071118

**Published:** 2016-07-19

**Authors:** Tao Yang, Huifen Huang, Fang Zhu, Qinlu Lin, Lin Zhang, Junwen Liu

**Affiliations:** 1 College of Food Science & Engineering, Central South University of Forestry & Technology, Changsha 410004, China; yangtao807@163.com (T.Y.); huanghuifen011@163.com (H.H.); lwsw01@163.com (F.Z.); linql0403@126.com (Q.L.); wolaitb@163.com (L.Z.); 2 Department of Histology and Embryology, School of Basic Medical Sciences, Central South University, Changsha 410013, China

**Keywords:** nanobiosensing, food safety analysis, function of nanomaterials

## Abstract

With increasing adulteration, food safety analysis has become an important research field. Nanomaterials-based biosensing holds great potential in designing highly sensitive and selective detection strategies necessary for food safety analysis. This review summarizes various function types of nanomaterials, the methods of functionalization of nanomaterials, and recent (2014–present) progress in the design and development of nanobiosensing for the detection of food contaminants including pathogens, toxins, pesticides, antibiotics, metal contaminants, and other analytes, which are sub-classified according to various recognition methods of each analyte. The existing shortcomings and future perspectives of the rapidly growing field of nanobiosensing addressing food safety issues are also discussed briefly.

## 1. Introduction

Food safety is a significant public concern, directly impacting human health worldwide. Contaminants, such as harmful bacteria, chemicals, natural toxins, or heavy metals in food can cause several diseases, including gastrointestinal, neurological, immunological diseases, multi-organ failure, and even cancers. Therefore, supervision and addressing the issues related to food safety need to exploit multifarious strategies to minimize the risk of contamination being transferred through the chain. Moreover, for contaminants generally present in trace quantities in food, qualitative approaches are less significant and positive/absence tests are sufficient. Hence, sensitive and quantitative techniques accompanying simple, rapid, and cost-effective approaches would be necessary to detect these trace substances. Traditionally, several technologies, such as enzyme-linked immunosorbent assay (ELISA), mass spectrometry (MS), chromatography, and capillary electrophoresis (CE) have been extensively applied to develop different sensing techniques for the determination of food contaminants. Despite possessing the merits of sensitivity and accuracy, these technologies have many disadvantages, including complication in execution, are time-consuming, require expensive instrumentation and professional skills, which greatly limits them from broader applications. 

Biosensing, combining a biological component with a physicochemical detector, is an approach used to detect various analyte. The high sensitivity and specificity that come out of shapely specific recognition are the greatest advantages of biosensing. Advances in nanomaterials have facilitated development of biosensing for detection of hazards associated with foods [1,2,3], where application of nanomaterials in biosensing has several key advantages including (1) better target identification; (2) enhancement in signal output through rapid recognition; (3) increase in selectivity and sensitivity; and (4) decrease in analysis time. Different nanomaterials, including zero-dimensional (0D) nanoparticles (NPs, including nanodots), 1D nanorods (containing nanowires and nanotubes), 2D nanosheets, and even 3D metal organic frameworks (MOFs), have been effective in meeting the challenges to establish advanced nanobiosensing methods. Examples of these nanomaterials can be stratified into following categories: metallic NPs (nanoclusters, nanorods), metal compound nanomaterials, carbon materials, non-metallic nanomaterials, nanostructures, and composite nanomaterials. Among these materials, graphene (including graphene oxide (GOx)) and gold NPs (AuNPs) have been found to have more applications so far. Graphene is a type of 2D carbon material comprising a single layer of sp^2^-hybridized carbon atoms that covalently forms a flat hexagonal lattice [4]. AuNPs possess high surface-to-volume ratio and unique optoelectronic properties that can be readily regulated by altering the size, shape, or surrounding environment and, thus, making them excellent scaffolds for application in novel chemical and biological sensors [5,6].

This review discusses the recent advances (2014 to present) in nanomaterial-based biosensing methods for addressing the food safety issue. We will begin with a brief discussion on various functions of nanomaterials in food safety risk analysis as well as different functionalization methods of nanomaterials, followed by a detailed discussion on the applications of nanomaterials in biosensing focusing on some significant advances. Especially, one type of analyte will then be subdivided into several subcategories according to its various recognition elements. In addition, the review summarizes the limitations of current nanobiosensing detection systems and proposes a few suggestions for prospective development.

## 2. Different Functional Roles of Nanomaterials in Food Safety Analysis

Nanomaterials can play various roles in different nanobiosensing-based methods. They may function as a carrier or enhancer, or as a catalyst, reporter, quencher, or separator. 

*Carrier.* Nanomaterials (such as graphene and metallic NPs), owing to their relatively large surface area and porous nature, have usually been used as a carrier to load multifarious substances [7,8,9]. For example, GOx has been utilized as a nanocarrier to load both AuNPs-coated SiO_2_ nanocomposites (Au@SiO_2_) and thionine [10], electrodeposited nanoAu can act as the carrier for fluorescence-decorated DNA probe [11], and MOFs can encapsulate Eu^3+^ cations into their pores [12]. Furthermore, AuNPs are often utilized as the supporting materials of silver enhancement [13].

*Enhancer.* An enhancer is a nanomaterial that, because of the high surface-to-volume ratio and high conductivity, can be used to enhance the physical signal of biosensing. Metal NPs and carbon materials have commonly been used in electrochemical sensors to enhance electrochemical signal and sensitivity [6,14,15,16]. Nanomaterials have also been reported for enhancing sensitivity in the sensors based on surface plasmon resonance (SPR), quartz crystal microbalance (QCM, mass effect), and metal-enhanced fluorescence (MEF effect) [17,18,19]. Inherent low-efficiency inelastic photon scattering severely limits application of surface-enhanced Raman spectroscopy (SERS) in sensitive detection of analytes; however, plasmonic NPs can significantly improve Raman scattering intensity up to billions of times, thereby increasing sensitivity, i.e., lowering the limit of detection (LOD) [20,21,22,23].

*Catalyst.* Many nanomaterials exhibiting high peroxidase activity, such as noble metal NPs [24,25,26], metallic oxide NPs and composite NPs [27,28], have been reported to detect food contaminants. Horseradish peroxidase (HRP) mimicking NPs can catalyze the degradation of H_2_O_2_, thus leading to either direct generation of changed electric signal or indirect oxidization of hydroquinone (electrochemistry), luminol (chemiluminescence), 3,3′,5,5′-tetramethylbenzidine (TMB), or 2,2′-azino-bis(3-ethylbenzothiazoline-6-sulphonic acid) (ABTS, colorimetric methods). 

*Reporter.* A reporter nanomaterial is a nanomaterial that can be used as electrochemical, colorimetric, fluorescent, or other types of signal molecule. Metal NPs [29], metallic oxide NPs [30,31] and QDs [32,33] are known to function as electrochemical reporter (stripping voltammetry). On the other hand, metal nanoclusters [34,35], QDs [36,37] and up-conversion NPs [38] can emit fluorescence that can influenced by quencher, change in structure or environment [39]. The aggregation of metal NPs (especially, AuNPs and AgNPs) of appropriate sizes induces interparticle surface plasmon coupling, generating visible color change—from red to blue for AuNPs and from yellow to brown for AgNPs) [40,41].

*Quencher.* Fluorescence or electrogenerated chemiluminescence (ECL) quenching is a commonly observed consequence when fluorescent substances or luminophores are appended onto/near some nanomaterials. Quenching occurs when the emission spectrum of chromophore overlaps with the surface plasmon band of nanomaterials, known fluorescence resonance energy transfer (FRET) or inner filter effect (IFE) [38,42,43]. Interestingly, the small AuNPs exhibit higher quenching efficiency than the large AuNPs [6,44].

*Separator.* Magnetic NPs (MNPs), commonly consisting of magnetic elements such as Fe, Ni, and Co and their chemical compounds, have been used for pretreatment of different materials as well as for separation of target analytes from complicated compositions. Studies have shown importance of MNPs in rational nanobiosensing design [45,46].

Although this section discusses separately individual functions of nanomaterials in sensors designed to detect trace food contaminants, nanomaterials can also function in multimodal way, i.e., one type of nanomaterials may involve in more than one function (Table 1). For example, graphene not only works as a carrier (such as for loading DNA), it also acts as a quencher (such as for quenching the fluorescence of the QDs labeled with DNA) [47]. Trifunctional Au doped Fe_3_O_4_ (Au@Fe_3_O_4_) NPs are another example of NPs those works in multimodal way—while Fe_3_O_4_ core involves in magnetic separation, gold shell takes part in dual function, carrying aptamer (oligonucleotide or peptide that specifically bind to a target molecule), and catalyzing H_2_O_2_ [28].

### Functionalization of Nanomaterials

Functionalization is one of the approaches that prepare nanomaterials suitable for a definite function or purpose. Nanomaterials can be functionalized through various routes, non-covalent or covalent to obtain complex hybrid systems. Non-covalent interactions include electrostatic adsorption (e.g., multi-charged AuNPs) [48], π-π stacking (e.g., carbon nanotubes and graphene with delocalized π-bond) [47], embedding [16,49], and specific affinity interactions (e.g., aptamer-target, biotin-streptavidin, and antigen-antibody) [50,51]. Covalent interactions play increasingly important role in functionalization of nanomaterials. Amino-carboxyl compounds (based on 1-ethyl-3-(3-dimethylaminopropyl)-carbodiimide/*N*-hydroxysuccinimide (EDC/NHS)) are most commonly used to functionalize variety nanomaterials [52,53], while metal-S is prevalent to functionalize metal NPs and QDs [23,54]. Other approaches of functionalization include metal-ligand [55], efficient click chemistry [56], and SN2 mechanism [57]. 

## 3. Recent Development in Nanobiosensing for Food Safety Analysis

This section focuses on the recent developments in the field of nanobiosensing for sensitive detection of food contaminants. We have divided this section into six sub-sections based on the type of contaminant detected by those nanobiosensing. Each type of contaminant were then classified to several subcategories based on the identification methods towards analytes.

### 3.1. Pathogens

Several foodborne infections are commonly caused by microorganism such as bacteria, viruses, and protozoa. Counting with colony-forming units (CFU) is the traditional and culture-based method for detecting such substances; however, this method is time-consuming, expensive, as well as laborious [58]. In addition, not all microbes can be cultured under laboratory conditions, thereby increasing the demand for non-culture-based techniques. Nanobiosensing with high sensitivity and selectivity are good for initial screening of food microorganisms and could be a better alternative to colony counting [5].

**Figure 1:**
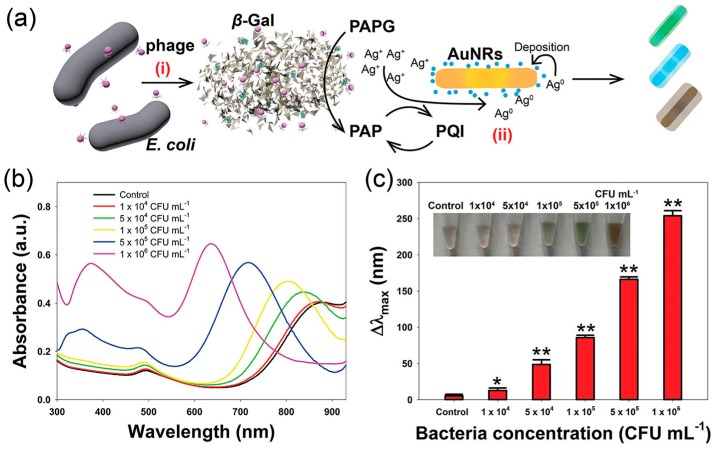
(**a**) Schematic illustration of the enzyme-induced metallization colorimetric assay for the detection of *E. coli* cells; (**b**) UV–vis absorption spectra of the colorimetric assay toward various E. coli concentrations; (**c**) The blue shift in the longitudinal LSPR peak toward various E. coli concentrations (inset: the corresponding photographs). Reprinted with permission from [78]. Copyright (2016) Wiley-VCH Verlag GmbH and Co. KGaA, Weinheim.

(1) Recognized by complementary DNA (cDNA). One of the detection routes for microbial pathogens involves analyzing its genomic DNA (gDNA) [10,59,60,61,62] which can be specifically recognized by its cDNA. Since only a trace amount of target DNA is present in microbial pathogens, nanomaterials and amplification techniques (such as polymerase chain reaction (PCR, a non-isothermal and enzymatic process based on using DNA polymerase to synthesize new strands complementary to the offered template strand), rolling circle amplification (RCA, an isothermal and enzymatic process in which long single-stranded DNAs (ssDNA) are synthesized on a short circular ssDNA template by using a single DNA primer), DNAzyme) are concurrently recruited to amplify target DNA or signal. Recently, a metallic nanowire based electrical *Escherichia coli* (*E. coli*) genomic DNA detection method has been developed using RCA to generate long ssDNA with abundant repetitive sequences [59]. DNA modified AuNPs of 10 nm diameter is aligned along long ssDNA via DNA hybridization, followed by enhancing conductivity of AuNPs string using silver or gold solutions to form wide silver or gold nanowires, resulting a high signal-to-noise ratio and low limit of detection (LOD) towards *E. coli* gDNzA. In addition, GOx-HRP mimicking DNAzyme nanocomposites, AuNPs-magnetic Fe_3_O_4_ NPs, and DNA functionalized AuNPs-asymmetric PCR system have been employed for the detection of gDNA of microbial pathogens [10,60,61]. However, this strategy is hampered by cumbersome pretreatment of pathogen and extraction of gDNA.
(2) Recognized by antibody. Antibodies with affinity towards the pathogens (immunologic approach) is a more convenient approach than analysis of gDNA [63,64,65,66,67]. A novel, sensitive, amplified detection of *E. coli* O157:H7 in food at real-time has been developed based on Pt–Au bimetal NPs with peroxidase activity using immunochromatographic assay (ICA) [27]; *E. coli* O157:H7 is one of the most notorious pathogens with low infectious dose commonly found in beef, raw milk, and vegetables. Indirect immunofluorescence assay, designed using FITC (fluorescein isothiocyanate)-doped silica NPs synthesized by W/O microemulsion method, demonstrated rapid detection of *E. coli* O157:H7 in beef [53]. In addition, polydiacetylene liposomes incorporated with antibody can be used for specific detection of *Salmonella*; the using of small liposomes can help in enhancing sensitivity [68]. Portable and automated paper-based detection methods are being rapidly developed in recently [69]. Merkoçi and co-workers have invented a lateral flow immunoassay for highly sensitive paper-based *E. coli* detection [70]. This design includes CdSe@ZnS QDs decorated with antibody (Ab-QDs) and GOx as photoluminescent probes and revealing-agent. The proposed device demonstrates highly specific and sensitive performance, detecting pathogen 10 CFU·mL^−1^ in standard buffer and 100 CFU·mL^−1^ in bottled water and milk. The similar portable and paper-based principle has been adopted using Pt–Au bimetal NPs and TMB as catalyst and colorimetric substrate, respectively [27], therefore, the pathogen detection can directly be observed by naked eyes. This proposed device exhibits a lower LOD of 100 cells/mL, which is 1000-fold lower than the AuNPs-based colorimetric method.
(3) Recognized by aptamer. Using antibodies as a part of a sensing system has some serious drawbacks such as rigorous production and purification processes and limited applicability (not work in harsh conditions, e.g., high temperature) [71]. These weaknesses can be neglected when using aptamer as recognition element. Many aptasensings based on nanomaterials (MNPs, silver NPs, nanorods, carbon quantum dots, and so on) have been designed for the quantification of microbial pathogen in various real samples [23,72,73,74,75]. Employing aptamer-conjugated fluorescent NPs and multicolor upconversion NPs as reporters, the LODs for *Staphylococcus aureus*, *Vibrio parahemolyticus*, and *Salmonella typhimurium* can lower to 25, 10, and 15 CFU·mL^−1^, respectively [76,77]. Alternatively, monitoring and measuring beta-galactosidase (β-gal) activity is another approach to detect *E. coli*. In the presence of β-gal released from *E. coli*, the substrate *p*-aminophenyl β-d-galactopyranoside is hydrolyzed to produce *p*-aminophenol. Reduction of Ag^+^ by *p*-aminophenol generates a silver shell on the surface of gold nanorods (AuNRs), resulting in the blue shift of the longitudinal localized surface plasmon resonance peak and multicolor change of the solution from light green to orange-red (Figure 1) [78].

### 3.2. Toxins

Due to improper storage, agricultural produce and animal feedstuffs are easily contaminated with toxins produced by filamentous fungi or bacteria as their secondary metabolites. For example, mycotoxins contaminate about a quarter of worldwide grains [79]. Even a trace quantity of toxin can cause serious health problems including nephritic, hepatic, nervous diseases, carcinogenicity, or even death [80,81]. Therefore, the detection and prevention of foodborne toxins are of prime importance to maintain a healthy society. Compared to detecting producing cells, detecting toxins show several advantages, such as no requirement of cultivation, relative high analyte concentration (hence, more sensitive), and undemanding detection environment. Nanomaterials show great potential to be incorporated in diverse biosensing strategies for the rapid, sensitive, and specific detection of contaminants over the existing conventional methods.

**Figure 2:**
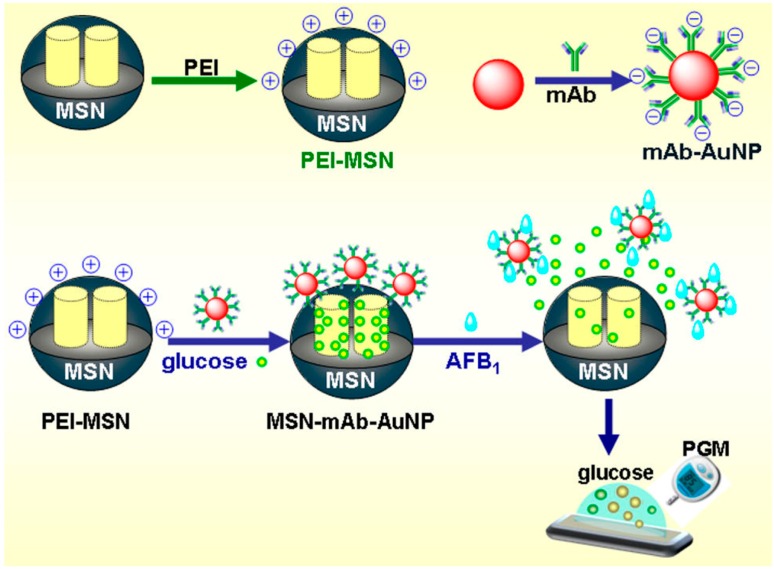
Schematic illustration of PGM-based immunosensing protocol using mAb-AuNP-gated PEI-mesoporous silica NPs loading with glucose. Reprinted with permission from [8]. Copyright (2014) American Chemical Society.

(1) Recognized by antibody. The majority of nanobiosensing techniques have been developed based on immunoassay. Tang et al. have developed an antibody-functionalized mesoporous carbon (MSC) NPs-based competitive-type biosensor for the detection of AFB1 (aflatoxin B1, classified as the first class carcinogen by WHO) [82] in peanuts. Recognition of AFB1 by antibody on MSC results in a departure of thionine—MSC from the electrode accompanying a decrease of current signal. Another competitive immunosensing strategy for the detection of AFB1 in peanut using mesoporous silica nanomaterial loaded with glucose and AuNPs as a lock (Figure 2) [8]. Interestingly, this low-cost, sensitive immunosensing platform can also be used with a portable personal glucometer (PGM) as the readout device [83]. The immune displacement reaction can open the lock and release glucose from the mesoporous silica to the solution, which can then be assayed by PGM. Other NPs, such as QDs, MNPs, and GOx, have also been used to develop nanobiosensors to detect toxins, including ochratoxins, aflatoxins, and deoxynivalenol (DON) in crops [52,84,85].
(2) Recognized by aptamer. Another significant mechanism is the interaction of a toxin with its aptamer. Ochratoxin A (OTA) was the first mycotoxin targeted by aptamer-based assay in 2008. Since then, several nanomaterials and aptamer-based methods have been developed. Recently, a novel strategy based on fluorescent nitrogen-doped carbon dots (N,C-dots) on AuNPs have been proposed for the detection of AFB1 in peanut and corn samples [86]. The chemically-inert N,C-dots provides excellent resistance to photobleaching. This N,C-dots/AuNPs-based aptasensor shows high selectivity against other normally-coexisted mycotoxins, such as OTA, DON, fumonisin B1, and zearalenone. Various metal compound nanomaterials, involving iridium oxide NPs [87], AuNPs doped Fe_3_O_4_ NPs [28], CdTe QDs-GOx [47], nanoceria tagged GOx [88], silver nanoclusters (AgNCs) [89] and have also been used to assay toxins. Nonetheless, the association constants of small molecules with their aptamers are low in general; therefore, to obtain a lower LOD, various amplification methods have been employed. Wei et al. have used GOx and DNase I to achieve target recycling, resulting in high sensitivity in OTA detection with a LOD of 20 nM in real red wine samples [90]. Combining unique properties of QDs and MNPs with high efficiency of RCA amplification, an optimized detection for OTA can attain an ultra-low LOD of 0.13 ppt, a 10,000-fold improvement compared with the traditional methods [45].
(3) Others. In addition to being recognized by antibodies and aptamers, many other nanomaterial-based mechanism were reported. (a) Nano-extraction with mass spectrometry (MS) [91]. Utilizing magnetic separation properties of MNPs, a magnetic solid phase extraction of aflatoxins from liquid samples has been developed using polydopamine-coated MNPs as the adsorbent. Coupled with HPLC-MS/MS quantification, LOD of 0.0012 ng/mL for AFB1, AFB2, and AFG1, and 0.0031 ng/mL for AFG2 can be achieved [92]; (b) NPs based molecular imprinting. An electrochemiluminescence sensor, based on Ru(bpy)_3_^2+^-doped silica NPs combined with molecularly imprinted polymer, has exhibited efficient detection of OTA in corn with a LOD of 0.027 pg/mL [93].

### 3.3. Pesticides

To protect plants from damaging influences from insects, pests, fungi or weeds and to ensure good crop health, pesticides are used. Pesticides are a class of biocide containing harmful chemical substances. The commonly-used pesticides include organophosphorus, pyrethroids, carbamates, and organochlorines. Although pesticides have beneficial effects, high neurotoxicity, and widespread use of pesticides beyond permissible limit have become a matter of grave concern considering the harmful aftereffects of pesticides on environment, food safety, and health. The accumulation of pesticides in animals and humans leads to serious diseases or even death. Hence, appropriate measures should be taken to control the use of pesticide, making more stringent rules over the permissible limit.

**Figure 3:**
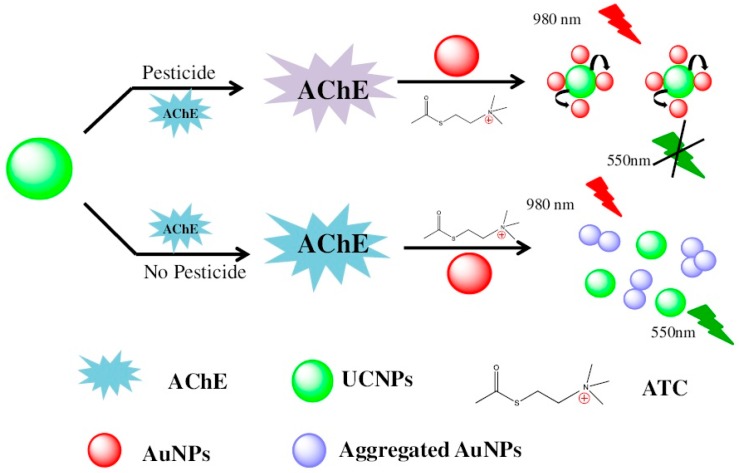
Schematic illustration of the UCNPs–AuNPs fluorescence assay for the detection of pesticides. Reprinted with permission from [38]. Copyright (2015) Elsevier.

(1) Enzyme inhibition by pesticide is the most mature and widely used technology for the rapid detection of pesticide residues. Organophosphorus compounds and carbamates can specifically inhibit the activity of acetylcholine esterase (AChE). Zhang and coworkers developed a novel nanobiosensing for organophosphorus pesticides. Thiocholine generation by AChE catalysis leads to the aggregation of AuNPs, resulting in the recovery of fluorescence resonance energy transfer (FRET) between AuNPs and NaYF4:Yb, upconversion NPs (Figure 3) [38]. However, AChE is unstable in solution. Immobilization of AChE in fenugreek hydrogel-agarose matrix with AuNPs results in high enzyme retention efficiency of 92% and a significantly prolonged half-life of the AChE (55 days) [94]. Apart from AChE, pesticides can also inhibit other enzyme activity such as trypsin and tyrosinase [95,96]. Trypsin easily hydrolyzes protamine covered on the surface of AuNPs, leading to fluorescence quenching of QDs. Conversely, the fluorescence could be recovered by adding methyl parathion as it inhibits trypsin activity [96].
(2) Organophosphorus hydrolase-based strategies involve direct detection mechanism than enzymes inhibition strategies. Organophosphorus hydrolase is a homodimeric enzyme that catalyzes the hydrolysis of organophosphorus pesticides. As uniform porous channels, large surface area and well-defined pore topology, ordered mesoporous carbons was used to immobilize cell surface-displayed organophosphorus hydrolase on electrode for direct determination of organophosphates such as paraoxon, parathion, and methyl parathion [97]. Similar direct detection method has also been developed using single-walled CNTs as carrier to support recognition material [7].
(3) Electrochemical and photochemical properties of pesticides themselves are commonly used to develop nanobiosensing. For example, omethoate, malathion, lindane, carbofuran, and carbaryl, etc. possess electrochemical properties. Therefore, nanobiosensors based on electrochemical analysis would be suitable for detecting those pesticides. Many such nanobiosensors, based on copper oxide nanowires-CNTs, AgNPs decorated polyaniline-nanocrystalline zeolite organic-inorganic hybrid material, cobalt oxide (CoO)-reduced GOx, zirconia-ordered macroporous polyaniline, and other nanosystems, have already been reported to improve the sensitivity [98,99,100,101,102]. In addition to electrochemical methods, a few NPs-enhanced SERS methods have been developed; however, low affinity limits the application of such methods. Such problems can be overcome by optimizing metal NPs, for example, the type, molecular linker, surface coverage, and laser excitation wavelength of NPs [103]. It is worth mentioning that, inspired by conductive ink pens for electronic devices on paper, Polavarapu et al. have developed a “pen-on-paper” approach for making SERS substrates [104]. The design involves employing an ordinary fountain pen filled with plasmonic inks comprising metal NPs with arbitrary size and shape; hence, no professional training is needed to manufacture SERS arrays on paper. This simple design lowers LOD of thiabendazole to 20 ppb. In spite of such progress in research, there is a limited translation of technology from laboratory to real life because of economic viability and operational simplicity.
(4) Recognized by antibody. In addition, immunoassay based nanobiosensing are most common for detecting pesticides in food [105,106,107]. The application of nanometal organic framework and other materials can greatly reduce the LOD [55]. As pesticides are known to impede certain photophysical as well as photochemical functions of nanomaterial, through specific recognition of pesticides by antibodies decorated on nanomaterial, several excellent phenomena have been discovered: pentachlorophenol obstructs electrochemiluminescence of Au nanoclusters/graphene hybrid [108], acetamiprid decreases enhanced photocurrent produced by electron donor of quercetin in Co-doped ZnO diluted magnetic semiconductor, thiram quenches blue luminescence of Cu^2+^ decorated NaYF4:Yb/Tm upconversion NPs fixed on filter paper (monitored by the smartphone camera through a self-written Android program) [109].

### 3.4. Antibiotics

Since the discovery and application of antibiotics, we have got a powerful weapon to combat against diseases and death. To enhance growth in animals, antibiotics are routinely used in husbandry. However, inappropriate use of antibiotics in animals will increase the incidence of antibiotic resistance and bring various side effects. The addition of some kinds of antibiotics into animal feed is strictly prohibited in some countries (e.g., enrofloxacin in USA). However, driven by the stakes, some farms illegally raise animals with excessive antibiotic for high profit, which will result in the antibiotic residues in produce, especially in meat and milk. Therefore, sensitive and infallible assays are imperative to assure the control of vestigial antibiotics in the products of farm animals (such as in milk and meats).

**Figure 4:**
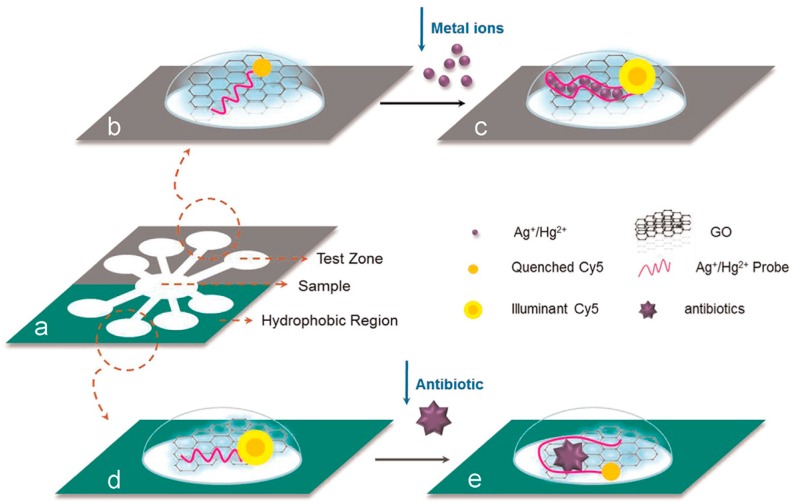
Schematic of the paper-based microfluidic device for multiplex chemical contaminants detection using ssDNA-functionalized GO sensors. Reprinted with permission from [111]. Copyright (2014) Elsevier.

(1) Recognized by aptamer. Aptamer-based nanobiosensing methods are the most common used for the detection of antibiotics. The upconversion NPs (anti-Stokes)-based aptasensor has shown good specificity towards kanamycin without being disturbed by other antibiotics [110]. Nanomaterials, such as GOx and AuNPs, are used as quenchers in assays based on aptamers of targets and fluorescence-labeled single-stranded DNA to detect antibiotics [111,112]. Simultaneous detection of multiple chemical contaminants in a food sample is a challenging task since each one functions in different microenvironment. Using GOx as quencher, Zuo et al. developed a low-cost paper based microfluidic device for detecting multiple chemical contaminants (antibiotics and heavy metal ions) simultaneously in food samples (Figure 4) [111]. Interestingly, other functions of antibiotics, for example, protecting nature (protecting AgNPs against salt-induced aggregation [113]) of kanamycin, can also be utilized to develop new biosensing methods.
(2) Recognized by antibody. Alternatively, immunization is another strategy to detect antibiotics, though it is not popular than the aptamer method. Metallic nanomaterials (gold nanoflower, AuNPs)-based electrochemical immunosensing methods have frequently been employed to assess chloramphenicol, ofloxacin, and tetracycline in multifarious foods, including milk, honey, and other samples [48,50]. In addition to electrochemistry, a competitive chemiluminescent immunoassay based on new luminol functionalized silver NPs was reported to determine chloramphenicol in milk and honey [114].
(3) Recognized by liposome. Liposomes were often used in molecular biology and pharmaceutics, but rarely used in other fields. Phospholipid liposomes containing R6G dyes on their surface have been utilized to develop a self-signaling sensing platform to detect neomycin—selective recognition of the target by phospholipid displaces R6G dyes from the surface and turns on fluorescence [115].

### 3.5. Metal Contaminants

Heavy metal ions, such as lead, mercury, cadmium, chromium, and arsenic, are hazardous, contributing to water and soil pollution [116,117,118,119,120,121,122]. Through water and soil, these metal residues reach daily foods. Heavy metals are known to cause irreversible changes in protein structures, affecting cell functions. Excessive intake of such substances can result in adverse health conditions including neurological disorders, renal degradation, and bone lesions [123]. 

The nanobiosensing methods for the detection of heavy metal ions can be divided into several subcategories according to recognition biomolecule. (1) Nucleotides. Chen has developed an AuNPs-based dual labeling colorimetric method for Hg^2+^ detection using a specific thymine–Hg^2+^–thymine (T-Hg-T) [57,124] as a recognition system and dual-labeling strategy for signal amplification; without using any instruments, they obtained an LOD of 0.025 nM, competitive to other rapid detection methods [125]. Using the same mechanism, a triple Raman label-encoded AuNPs trimer has been designed for simultaneous Hg^2+^ and Ag^+^ (cytosine–Ag^+^–cytosine, C–Ag^+^–C) [126] detections. The target ions aid in assembling AuNPs modified with different Raman labels, leading to different enhancements of Raman signal [127]; (2) DNAzyme: some heavy metal ions, such as Pb^2+^ and Ag^+^ [128], act as a co-factor of DNAzyme. Based on DNA-stabilized AgNCs (signal reporter) and DNAzyme (recognition group and amplifier), a label-free catalytic biosensing platform was developed for selective assay of Pb^2+^ [129]; (3) amino acid: several metal ions can specifically identified by amino acid because of the functional side chain (such as cysteine). Based on the graphene-enhanced electrochemical signal, the recognition of heavy metal ions (Cd^2+^ and Pb^2+^) can be characterized via the change of electrochemical signal [130]; (4) antibodies: in general, an antibody for ion is hard to screen. An antibody was obtained through the interaction of Cd^2+^ with EDTA, which was used to develop Cd^2+^ biosensing based on core-shell Au@Ag nanoparticles enhanced Raman scattering [131]; (5) others: a mechanism that arsenate displaces the chromophore-labelled DNA adsorbed on the surface of FeO NPs was reported [132].

### 3.6. Other Analytes

Some manufacturers and farms engage in food fraud for increasing profit margin, and such ill practices often lead to devastating results. Melamine, a chemical adulterant, is sometimes illegally added into milk powder to improve the apparent protein content [133]. A melamine aptamer derived from an abasic-site-containing triplex molecular beacon (tMB) has been proposed for sensitive recognition of melamine by integrating tMBs and fluorescent AgNCs [134]. Nitrite is harmful to humans and is widely used as an additive and preservative in food service industry. A biosensor towards nitrite was developed based on the direct electrochemistry of myoglobin on a reduced GOx-multi-walled CNTs-platinum NPs nanocomposite [135]. ZnO NPs are frequently considered to design biosensing strategies for the detection of bisphenol A, a ubiquitous environmental contaminant found in food products and aquatic ecosystems [136,137]. As H_2_O_2_ is a kind of unlawful decolorizer for food, a biosensing method towards H_2_O_2_ was developed based on the H_2_O_2_ enlarging AuNPs induced significant fluorescence quenching of BSA-AuNCs [42].

## 4. Conclusions and Future Perspectives

Table 2 lists several samples of the nanobiosensing reported in various literatures for food safety analysis. From all the above-mentioned literatures, AuNPs, QDs, and carbon nanomaterials are commonly used nanomaterials to develop nanobiosensing strategies. For one analyte, several nanobiosensing methods were developed to cater to different demands of food safety analysis. For the pursuit of sensitivity, fluorescent nanomaterials-based biosensing may be suitable. However, for the pursuit of portable approaches, electrochemical and colorimetric, rather than fluorescent nanomaterials-based, methods can be employed. 

The plenitude of the available literatures related to the application of nanomaterials (including NPs and nanostructures) in biosensing clearly indicates the successful utilization of nanomaterials in food safety analysis for pathogens, toxins, antibiotics, pesticides, metal contaminants, and other analytes. Of the large number of literature available, we have selected only those reports that either have substantial impacts on the progress of nanobiosensing or have genuine potential for future applications; for example, paper-based detection methods or portable devices. In spite of substantial progress, nanobiosensing for food safety analysis suffers from some limitations. (1) Diversity: complicated synthetic procedures, expensive reagents, and non-commercialization impede application of nanomaterials beyond AuNPs, QDs, and carbon nanomaterials. Therefore, simple, inexpensive and efficient synthetic methods might promote application of other nanomaterials; (2) universality: nanomaterials have yet to spread to all areas of food safety, such as the usage of DNA polyhedral and DNA origami nanostructures [138,139,140], synergy with bispecific monoclonal antibodies, and peptide aptamers [141,142]. Moreover, not all the food contaminants can be detected by nanobiosensing approaches because of the lack of recognition biomolecules; (3) practicability: some detection methods involve multi-step procedures, thus increasing analytical cost and difficulty in implementation. In addition, due to inherent complexity in real food samples, sample separation procedures are required to eliminate interferences. Rapid and cost-effective analytical methods integrating sample separation units may greatly improve practicability of nanobiosensing; (4) miniaturization: development of portable sensing kit would not only be cost effective but more convenient. Nanomaterials decorated screen-printed electrode and paper as well as development of new portable devices or employment of available devices (e.g., glucometer, piezometer, and smartphone) need to be explored to achieve miniaturization; and (5) application: the development of sensitive and specific biosensing devices is one of the approaches to verify food safety. The slow adoption of biosensors in the food industry is related to the need for AOAC approved methods or recognized by regulatory bodies. Therefore, introduction of new regulations might increase the demand for biosensing devices. In conclusions, for a scientist, research should be focused on the design and development of cost effective, sensitive, novel detection protocols by integrating advanced nanomaterials and nanotechnologies with traditional detection methods further.

## Figures and Tables

**Table 1:** Summary of types and functions of commonly used nanomaterials.

Category	Nanomaterial	Size * (Shape)	Main Function
Metallic nanomaterial	AuNPs	<100 nm (sphere)	Carrier, enhancer, reporter, quencher
	Silver NPs (AgNPs)	<100 nm (sphere)	Enhancer, reporter
	Platinum NPs (PtNPs)	<100 nm (sphere)	Catalyst
	Metal nanoclusters	<10 nm (sphere)	Reporter
Metal compound nanomaterials	Quantum dots (QDs)	1–10 nm (sphere)	Carrier, reporter
	Upconversion NPs	<100 nm (sphere)	Reporter
	Fe_3_O_4_ NPs	5–500 nm (sphere)	Separator
	CuO NPs	<100 nm (sphere)	Enhancer, catalyst
Non-metallic nanomaterials	SiO_2_ nanomaterials	Dozens of nm (sphere)	Carrier
	Polyaniline NPs	<100 nm (sphere)	Enhancer
Carbon materials	Graphene	Various (sheet)	Carrier, quencher
	Carbon nanotube (CNTs)	Various (tube)	Carrier, enhancer, quencher
	Carbon dots (C dots)	<10 nm (sphere)	Reporter
Nanostructures	DNA nanostructures	Various (polyhedron)	Carrier

* The size of nanomaterials depends on reaction conditions.

**Table 2:** Samples of nanobiosensing for the assay of food contaminants.

Type of Contaminant	Contaminant	Recognition Biomolecule	Nanomaterials Used	Functions of Nanomaterials	Detection Format	LOD	Ref.
Pathogens	*E. coli* O157:H7	cDNA	GOx, Au@SiO_2_	Carrier, enhancer	Electrochemical	0.01 nM	[10]
	*E. coli*	cDNA	AuNPs, Fe_3_O_4_	Reporter, seperator	Electrochemical	1.8 aM	[60]
	*C. sakazakii*	Antibody	Fe_3_O_4_, liposomes	Carrier, seperator	Fluorescent	10^3^ CFU/mL	[64]
	*Mycoplasma suis*	Antibody	AuNPs	Carrier, reporter	Colorimetric	100 ng/mL	[65]
	*S. aureus*, *V. parahemolyticus*, *S. typhimurium*	Aptamer	Upconversion NPs	Reporter	Fluorescent	25, 10, 15 CFU/mL	[76]
	*E. coli* BL21	β-galactosidase	Ag-AuNRs	Reporter	Colorimetric	10^4^ CFU/mL	[78]
Toxins	Aflatoxin B1	Antibody	AuNPs, SiO_2_	Carrier	Electrochemical	5 ppt	[8]
	Shiga-like toxin 1	Antibody	Al_2_O_3_-Fe_3_O_4_	Carrier, seperator	Mass spectrometry	44 pM	[91]
	Ochratoxin A	Aptamer	Au doped Fe_3_O_4_	Carrier, catalyst, seperator	Colorimetric	30 pg/mL	[28]
	Aflatoxin B1	Aptamer	N-doped C dots, AuNPs	Carrier, reporter	Fluorescent	16 pM	[86]
	Ochratoxin A	Aptamer	Nanoceria, GOx	Carrier, catalyst	Electrochemical	0.1 nM	[88]
Pesticides	Methyl parathion, monocrotophos, dimethoate	AChE inhibition	Upconversion NPs, AuNPs	Reporter, quencher	Fluorescent	0.67, 23, 67 ng/L	[38]
	Carbofuran, oxamyl, methomyl, carbaryl	AChE inhibition	AuNPs	Enhancer	Colorimetric	2, 21, 113, 236 nM	[94]
	Methyl parathion	Trypsin inhibition	QDs, AuNPs	Reporter, quencher	Fluorescent	18 ng/L	[96]
	Paraoxon, parathion methyl parathion	Organophosphorus hydrolase	Mesoporous carbon	Carrier	Electrochemical	9.0, 10, 15 nM	[97]
	Parathion	Antibody	nanoMOF	Carrier, enhancer	Electrochemical	0.1 ng/mL	[55]
Antibiotics	Kanamycin	Aptamer	Upconversion NPs, GOx	Reporter, quencher	Fluorescent	18 pM	[110]
	Streptomycin	Aptamer	AuNPs	Quencher	Colorimetric and fluorescence	73.1 nM, 47.6 nM	[112]
	Chloramphenicol	Antibody	AgNPs	Carrier, enhancer	Electrochemical	7.6 ng/mL^−1^	[114]
	Neomycin	Receptor	Liposome	Carrier	Fluorescent	2.3 nM	[115]
Metal ions	Hg^2+^, Ag^+^	Nucleotide	AuNPs	Carrier, reporter	SERS	8.4, 16.8 × 10^−12^ M	[127]
	Pb^2+^	DNAzyme	DNA-stabilized AgNCs	Reporter	Fluorescent	17 μM	[129]
	Cd^2+^, Pb^2+^	Amino acid	Graphene	Carrier	Electrochemical	0.45, 0.12 μg/L	[130]
	Ni^2+^	Antibody	Au@Ag core-shell NPs	Carrier, reporter	SERS	0.05 ng/mL	[131]
